# Microvascular Resistance Reserve Predicts Myocardial Ischemia and Response to Therapy in Patients With Angina and Nonobstructive Coronary Arteries

**DOI:** 10.1161/CIRCINTERVENTIONS.124.014477

**Published:** 2024-09-05

**Authors:** Aish Sinha, Haseeb Rahman, Ozan M. Demir, Kalpa De Silva, Holly P. Morgan, Matthew Emile LiKamWa, Matthew Ryan, Saad Ezad, Becker Al-Khayatt, Howard Ellis, Amedeo Chiribiri, Andrew J. Webb, Divaka Perera

**Affiliations:** British Heart Foundation Centre of Excellence and National Institute for Health Research Biomedical Research Centre at the School of Cardiovascular Medicine and Sciences, King’s College London, United Kingdom (A.S., H.R., O.M.D., H.P.M., M.E.L.K.W., M.R., S.E., B.A.-K., H.E., A.C., A.J.W., D.P.).; Guys’ and St. Thomas’ NHS Foundation Trust, London, United Kingdom (K.D.S., A.J.W., D.P.).

**Keywords:** coronary vessels, ischemia, microcirculation, microvascular angina, quality of life

Coronary microvascular disease is associated with an impaired quality of life and heightened risk of adverse cardiovascular outcomes. The hallmark of coronary microvascular disease is a diminished coronary flow reserve^1^ (CFR) and CFR <2.5 predicts maladaptive exercise physiology, ischemia on noninvasive assessment, and response to anti-ischemic therapy with excellent accuracy.^1,2^ However, CFR is affected by the conductance of both the epicardial and microvascular compartments. Microvascular resistance reserve (MRR) is a novel microcirculation-specific coronary physiological parameter^3,4^; however, the diagnostic and therapeutic thresholds in patients with angina and nonobstructive coronary arteries (ANOCA) are yet to be established.

We assessed the diagnostic accuracy of MRR at predicting abnormal exercise physiology, inducible ischemia, and response to anti-ischemic therapy in patients with ANOCA. We have previously published the inclusion criteria and study protocols,^1,2^ but in brief, we recruited patients with ANOCA who underwent simultaneous measurement of intracoronary pressure and Doppler flow velocity at rest and during hyperemia. The first cohort (n=85) underwent stress perfusion cardiac magnetic resonance (CMR) imaging and invasive coronary physiology assessment during supine bicycle exercise. Maladaptive exercise physiology was defined as impaired coronary perfusion efficiency during exercise, and myocardial ischemia was defined as endocardial-to-epicardial perfusion ratio <1.0 during hyperemia on CMR.^1^ The second cohort (n=87) underwent blinded coronary physiology assessment and were randomized into a crossover anti-ischemic therapy trial; response to therapy was defined as ≥60-second increment in exercise time from baseline.^2^ This study was approved by the National Health Service Research Ethics Committee (references 20/LO/1294 and 17/LO/0203), and written informed consent was obtained from all patients before enrollment. The data that support the findings of this study are available from the corresponding author upon reasonable request.

MRR was derived as (CFR/FFR)×(Pa_*rest*_/Pa_*hyper*_)

CFR indicates ratio of average peak velocity at hyperemia and rest; FFR, ratio of distal coronary pressure to aortic pressure during hyperemia; and Pa_*rest*_/Pa_*hyper*_, ratio of aortic pressure during rest and hyperemia.

Binary logistic regression was performed to test if MRR was associated with exercise physiology, inducible ischemia, and response to anti-ischemic therapy using univariable analysis and reported as odds ratio (95% CI). The Youden index in receiver operating characteristic curves was used to identify the optimal MRR threshold. The accuracy of optimal CFR and MRR thresholds was calculated as ([true positives+true negatives]÷[true positives+true negatives+false positives+false negatives])×100.

Of the 85 patients enrolled in the first cohort (age 57±10 years, females 78%), 45 had a CFR <2.5 and 40 had a CFR ≥2.5. FFR was 0.92±0.05 and MRR was 3.0±0.9. MRR was independently associated with maladaptive exercise physiology (odds ratio, 0.85 [95% CI, 0.78–0.93]; *P*<0.01) and ischemia on CMR (odds ratio, 0.94 [95% CI, 0.88–1.00]; *P*=0.04; per 0.1 unit increase in MRR). The optimal MRR threshold was 3.0 to predict maladaptive exercise physiology (sensitivity, 75% [95% CI, 60%–86%] and specificity, 95% [95% CI, 77%–100%]) and 3.2 to predict ischemia on CMR (sensitivity, 83% [95% CI, 70%–93%] and specificity, 56% [95% CI, 35%–76%]). CFR was numerically better than MRR at predicting maladaptive exercise physiology (area under the curve, 0.90 [95% CI, 0.82–0.98] versus 0.86 [95% CI, 0.77–0.94]; *P*=0.07), with diagnostic accuracies of 86% (95% CI, 75%–93%) and 80% (95% CI, 68%–88%) of the CFR <2.5 and MRR <3.0 thresholds, respectively. CFR and MRR predicted ischemia on CMR with similar accuracy (area under the curve, 0.70 [95% CI, 0.56–0.84] versus 0.70 [95% CI, 0.57–0.84]; *P*=0.85), with diagnostic accuracies of 70% (95% CI, 57%–80%) and 71% (95% CI, 59%–82%) of the CFR <2.5 and MRR <3.2 thresholds, respectively (Figure).

**Figure.**
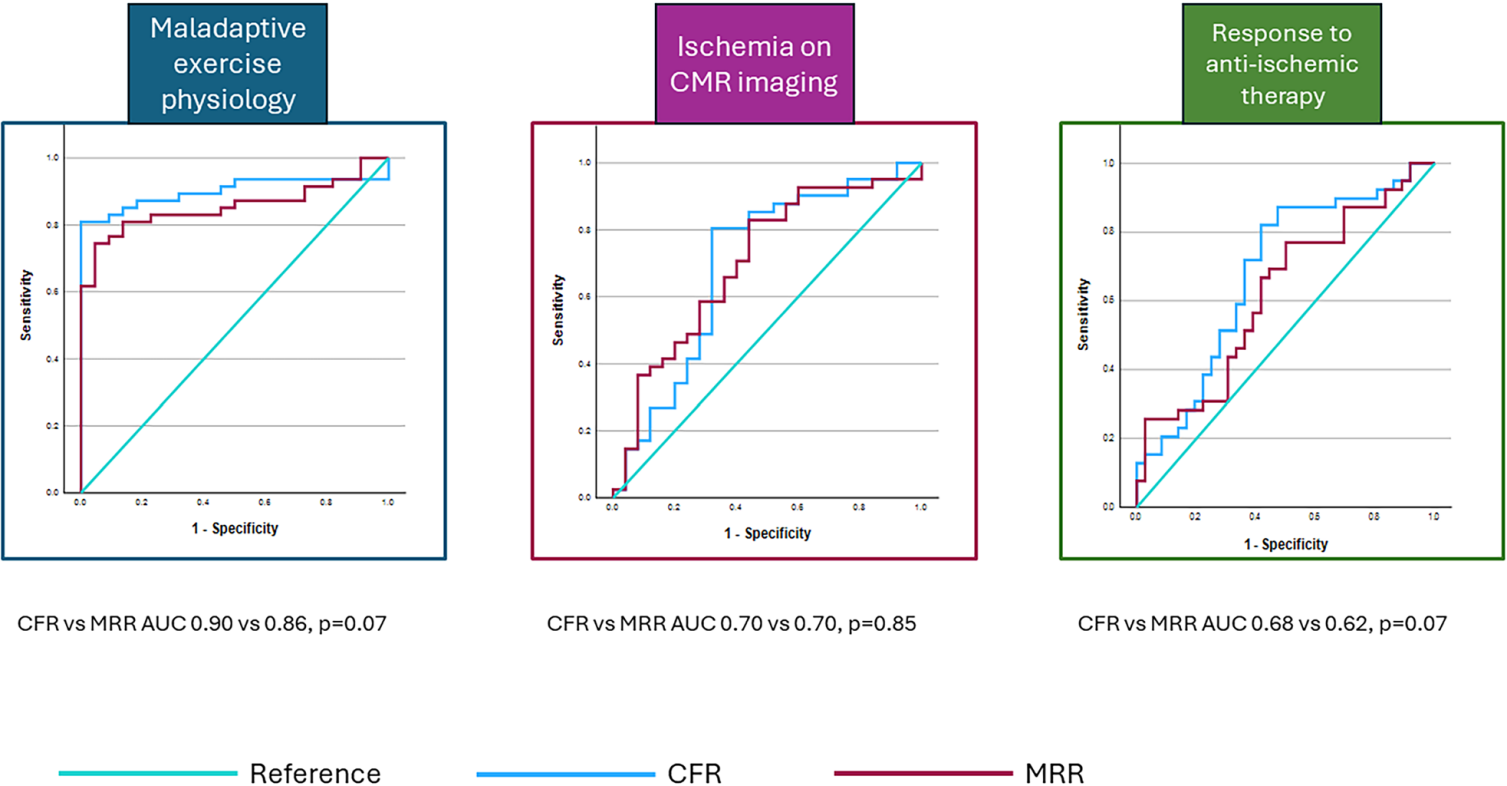
**Receiver operating characteristic curves comparing the ability of coronary flow reserve (CFR) and microvascular resistance reserve (MRR) at predicting maladaptive exercise physiology, ischemia, and response to therapy.** AUC indicates area under the curve; and CMR, cardiac magnetic resonance.

Of the 87 patients enrolled in the second cohort (age 61±8 years, females 62%), 57 had a CFR <2.5 and 30 had a CFR ≥2.5. FFR was 0.92±0.05 and MRR was 2.7±0.7. MRR was independently associated with a response to anti-ischemic therapy (odds ratio, 0.93 [95% CI, 0.87–1.00]; *P*=0.04; per 0.1 unit increase in MRR). The optimal MRR threshold to predict a response was 2.9 (sensitivity, 77% [95% CI, 61%–89%] and specificity, 50% [95% CI, 33%–67%]). CFR was numerically better at predicting response to anti-ischemic therapy than MRR (area under the curve, 0.68 [95% CI, 0.56–0.81] versus 0.62 [95% CI, 0.50–0.75]; *P*=0.07), with diagnostic accuracies of 68% (95% CI, 57%–78%) and 64% (95% CI, 52%–75%) of the CFR <2.5 and MRR <2.9 thresholds, respectively (Figure).

Our study demonstrates, for the first time, that MRR predicts maladaptive exercise physiology, inducible ischemia, and response to anti-ischemic therapy in patients with ANOCA. Notwithstanding the fact that MRR is a continuous variable, the diagnostic and therapeutic thresholds we have found could be adopted in clinical practice and future research studies. These thresholds are very similar to that which was recently reported as predictive of adverse outcomes in allcomers with ischemic heart disease (including epicardial and/or microvascular disease).^5^ MRR was not superior to CFR in patients with ANOCA, but, as MRR is proportional to CFR and inversely proportional to FFR, the most impactful utility of MRR may be in patients with concomitant epicardial and microvascular disease.^4^ MRR is a metric that relies on measurement of coronary flow as well as pressure; while we used Doppler to estimate flow in our study, continuous intracoronary thermodilution may be an attractive alternative technique, especially as it has less interoperator variability and can be performed without pharmacological hyperemia.

## ARTICLE INFORMATION

### Acknowledgments

The authors offer appreciation to all study investigators, clinical site staff, and study participants.

### Sources of Funding

The trial was funded by grants from the Medical Research Council (MR/T029390/1) and British Heart Foundation (FS/16/49/32320).

### Disclosures

None.
